# 584. Microbiological Outcomes of Culture-Negative Blood Specimens Using 16s rRNA Broad-Range PCR Sequencing: a Retrospective Study in a Canadian Province from 2018 to 2022

**DOI:** 10.1093/ofid/ofad500.653

**Published:** 2023-11-27

**Authors:** Anthony Lieu, Luke B Harrison, Josée Harel, Matthew Cheng, Marc-Christian Domingo

**Affiliations:** Stanford Health Care, Stanford, California; McGill University Health Centre, Montreal, Quebec, Canada; Institut de santé publique du Québec, St-Anne-de-Bellevue, Quebec, Canada; McGill University Health Centre, Montreal, Quebec, Canada; Laboratoire de Santé Publique du Québec, Montreal, Quebec, Canada

## Abstract

**Background:**

Broad-range bacterial PCR sequencing (BRBPS) has emerged as a novel tool to detect fastidious organisms. While its utility has been characterized in different specimen types, its role in culture-negative blood specimens remains poorly understood.

**Methods:**

We reviewed all clinical specimens sent for culture-negative blood BRBPS (blood, serum and blood culture bottles) to the Laboratoire de santé publique du Québec, the reference laboratory for a large Canadian province, from May 2018 to November 2022. Sanger sequencing of the amplified 16s rRNA gene was performed in all PCR-positive specimens. Data were extracted from the laboratory information system, and the analysis was restricted to the first specimen per patient. Microbiological outcomes were categorized as interpretable sequence, uninterpretable sequence, or negative PCR result. Interpretable sequences were identified and then classified based on the National Healthcare Safety Network database (NHSN; Table 1) and microbiological characteristics.

**Table 1.**
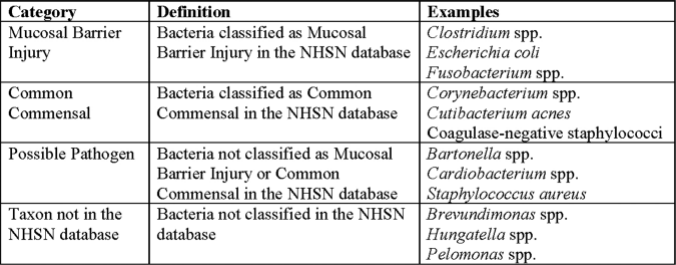
Definitions of National Health Safety Network (NHSN) Classification and Examples

**Results:**

A total of 1199 blood specimens were analyzed using BRBPS from 852 unique patients. Of these, there was no PCR amplification in 152, amplification with uninterpretable sequences in 445 and an interpretable sequence in 255 specimens (Figure 1). Blood specimens received at room temperature, in blood culture bottles, or with positive gram stain were more likely to yield interpretable sequences (Table 2). We identified 174 patients with BRBPS results suggestive of organisms associated with mucosal barrier injury (n=89) or possible pathogens (n=85), summarized in Figure 2. In contrast, 75 patients had results suggestive of contamination from common commensal organisms (n=44) or taxa not in the NSHN database (n=31).

**Figure 1.**
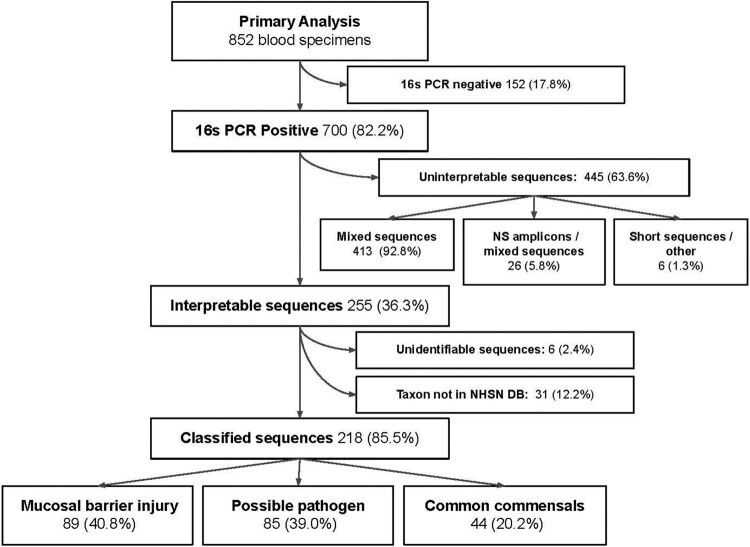
Flowchart of the analysis from primary specimens to those specimens with successful amplification of 16s rRNA

Classification of interpretable sequences using the National Healthcare Safety Network database classification of microorganisms.

**Table 2.**
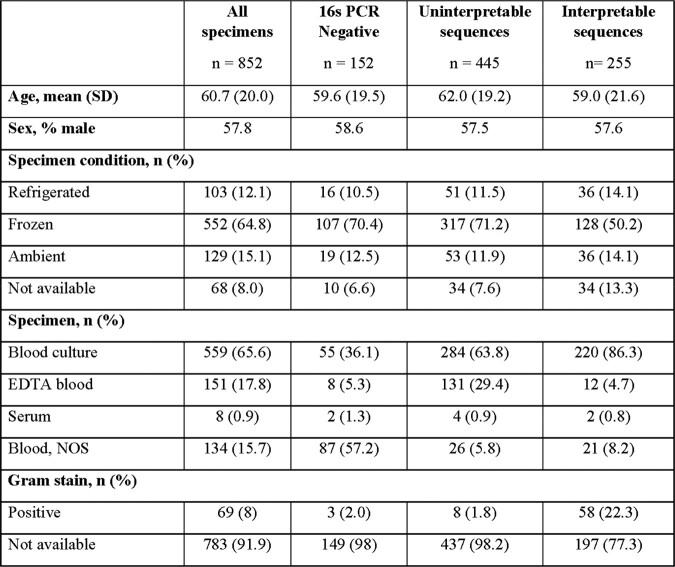
Characteristics of BRBPS of the 16s rRNA on Culture-Negative Blood Specimens

**Figure 2.**
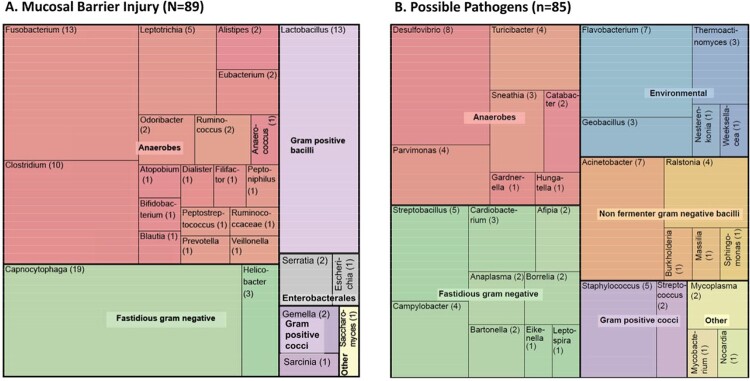
Tree Map of Mucosal Barrier Injury Organisms and Possible Pathogens by Genera

Mucosal barrier injury (A) and possible pathogens (B) are categorized based on the National Healthcare Safety Network database. Microorganisms are colour classified based on microbiological characteristics.

**Conclusion:**

Our findings demonstrate the potential utility of BRBPS in blood specimens from culture-negative patients, particularly infectious syndromes caused by fastidious gram-negative bacteria associated with animal or arthropod exposures or anaerobic bacteria. However, the frequent recovery of commensal and environmental organisms argues for careful and judicious use. Additional technical optimization is likely required to improve diagnostic yield, particularly with mixed sequences.

**Disclosures:**

**Matthew Cheng, MD**, Amplyx Pharmaceuticals: Grant/Research Support|AstraZeneca: Advisor/Consultant|AstraZeneca: Honoraria|Cidara Therapeutics: Grant/Research Support|GEn1E lifesciences: Advisor/Consultant|GEn1E lifesciences: Stocks/Bonds|Kanvas Biosciences, Inc.: Board Member|Kanvas Biosciences, Inc.: Pending patents|Kanvas Biosciences, Inc.: Ownership Interest|Merck: Honoraria|nomic bio: Advisor/Consultant|nomic bio: Stocks/Bonds|Pfizer: Honoraria|Scynexis Inc.: Grant/Research Support|Takeda: Advisor/Consultant|Takeda: Honoraria

